# Comparative study of the elimination of copper, cadmium, and methylene blue from water by adsorption on the citrus *Sinensis* peel and its activated carbon

**DOI:** 10.1039/d1ra08997h

**Published:** 2022-03-31

**Authors:** Wassim El Malti, Akram Hijazi, Zahraa Abou Khalil, Zahraa Yaghi, Mohamad Kazem Medlej, Mohamad Reda

**Affiliations:** College of Engineering and Technology, American University of the Middle East Kuwait Wassim.elmalti@aum.edu.kw; Research Platform for Environmental Science (PRASE), Doctoral School of Science and Technology Lebanon

## Abstract

The accumulation of heavy metals and dyes in wastewater is a persistent environmental threat with serious hazards consequences affecting all living organisms. Their removal has become a challenging environmental requirement. Adsorption using agricultural waste is one of the cost-effective removal techniques in which the biomass can be valorized. In this study, two adsorbents were prepared and compared in removing copper, cadmium, and methylene blue from water: citrus *Sinensis* peel (CP) and its activated carbon (AC). Many physical and chemical properties of the prepared adsorbents were investigated using several techniques. Various operational parameters such as initial adsorbate concentration, contact time, pH, adsorbent mass, and temperature were examined. The optimum uptake of Cd, Cu, and MB was obtained after 2 h contact time by using 0.25 g of adsorbent and 400 mg L^−1^ metal ions or 100 mg L^−1^ MB initial concentration at pH 5 (for metal ions only) and temperature of 25 °C. Slight superiority for the CP was seen. Furthermore, isothermal models were resolved in all the studied cases. Unlike for MB, the Langmuir model is more applicable for the adsorption of the cations on both adsorbents with maximum adsorption of 80 mg g^−1^ of Cd(ii) on CP. Finally, the adsorbents achieved good reuse performance, especially for CP which can be used up to 4 times to remove the metal ions, proving that they are low-cost and environmentally friendly materials able to remove inorganic and organic contaminants from water.

## Abstract 2

The accumulation of heavy metals and dyes in wastewater is a persistent environmental threat with serious hazards consequences affecting all living organisms. Citrus *Sinensis* peel and its activated carbon particles effectively remove Cu(ii), Cd(ii), and MB from water.

## Graphical abstract

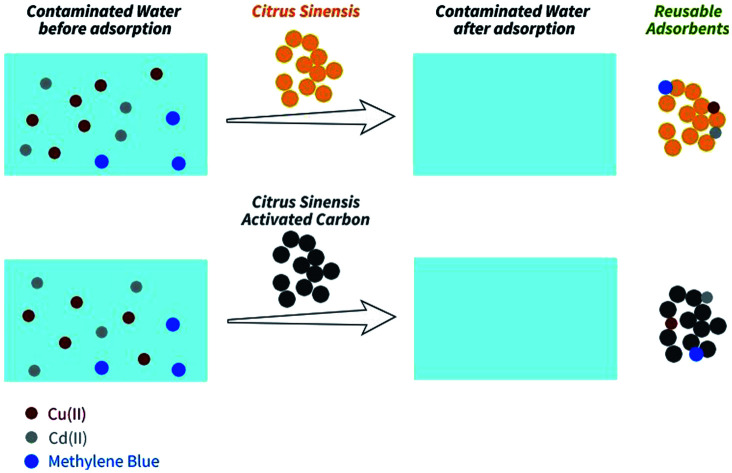

## Introduction

The excessive emission of organic and inorganic pollutants in water by various industrial, medical, and agricultural activities is a critical global environmental problem.^1^ Inorganic contaminants, mainly heavy metals such as copper (Cu) and cadmium (Cd), are not susceptible to biological degradation and accumulate in living tissues through the food chain, causing harmful and severe health issues.^2^ Similarly, the released organic contaminants, such as dyes, can accumulate in the environment generating aesthetic pollution and causing a detrimental effect on photosynthesis.^3^ Furthermore, methylene blue (MB) ingestion can produce severe health conditions.

Several conventional techniques were used to remove heavy metals and dyes from wastewater, including precipitation, ion exchange, membrane separation, coagulation–flocculation. However, most of these techniques encounter drawbacks such as high cost, production of sludge, low selectivity, and other operational complications.^4,5^

Due to its easy handling and simplicity in design, the adsorption process has been widely developed in treating wastewater for heavy metal ions and dyes removal.^6^ Many studies consisting of implementing highly efficient, eco-friendly, and low-cost adsorbents were reported in the literature.^7^ Recently, with the significant increase in environmental awareness, modified or unmodified agriculture waste and its activated carbon have been valorized and used as adsorbents for removing organic and inorganic contaminants from wastewater.^8^ Banana peel, papaya wood, waste tea leaves, spent coffee ground,^9^ coconut husk,^10^ pea shells activated carbon, and palm fruit activated carbon are examples of adsorbents that showed encouraging results in the sorption process. Table 1 includes significant results obtained by removing Cd, Cu, and MB from water using a few of the listed agriculture waste and its activated carbon in the literature. The efficiency of the metal uptake and the adsorption of the dyes depended on the surface properties of the adsorbent.

**Table 1:** Adsorption capacity (%) of some agriculture waste and its activated carbon reported in the literature

Adsorbent	Adsorbate	Adsorption capacity (%)	Reference
Banana peel	Cd	89.2%	11
	Cu	88.0%	12
	MB	90.0%	13
Papaya wood	Cd	94.9%	14
	Cu	97.8%	14
Waste tea leaves	Cd	99.5%	15
Coconut husk	Cd	95.2–98.8%	16
	Cu	75.0–98.5%	16
Pean shells activated carbon	Cd	95.6%	17
	Cu	96.4%	17
	MB	99.7%	18
Palm shells activated carbon	Cd	90.0%	19
	Cu	95.0%	19
	MB	97.1%	20

Usually, agriculture waste includes diverse organic compounds, such as phenols, cellulose, hemicellulose, and pectin, and has several functional groups on its surface. Hydroxyl, carboxyl, and carbonyl groups are the main surface functional groups with a good tendency for metal ion complexation and electrostatic interactions.^21^

Citrus *Sinensis* is one of the most fruit collections globally available, popular, and consumed. It grows worldwide in more than 140 countries in tropical and subtropical regions, with an annual production of approximately 110 million tons.^22,23^ Citrus *Sinensis* peels (CP) represent almost 44% of the fruit body, producing an enormous by-products mass which is usually discarded as waste. Thus, these peels represent an excellent potential to be valorized. The activated carbon (AC) derived from the peels can be produced by pyrolysis and chemical activation using an oxidizing agent, such as hydrogen peroxide (H_2_O_2_), penetrating the interface through the CP porous surface.

This work aimed to compare the adsorption performance of the valorized CP and its AC in removing Cu(ii), Cd(ii), and MB. The AC was synthesized by pyrolysis of CP and activation using an H_2_O_2_ aqueous solution. Different chemical and physical properties of both adsorbents were determined using several techniques, including particle size analysis, scanning electron microscopy (SEM), Brunauer–Emmett–Teller (BET), energy-dispersive X-ray analysis (EDS), Fourier transform infrared spectroscopy (FTIR), and zeta potential. A batch of adsorption experiments was performed to investigate the adsorption performance by varying diverse experimental parameters such as initial adsorbate concentration, contact time, pH, adsorbent mass, and temperature. Langmuir and Freundlich adsorption isotherms were built at optimum conditions and sufficient contact time to understand the interaction of the different studied adsorbates with the implemented adsorbents. Finally, reusability tests were conducted to examine the regeneration performance of both implemented adsorbents.

## Materials and methods

### Preparation of citrus *Sinensis* peel (CP) powder

The citrus *Sinensis* peel was collected from a local Lebanese region, washed with deionized water, cut into small pieces, then left to dry at 25 °C for 48 hours. After complete drying, they were ground to 1 mm particles, and the resulting powder was washed with deionized water and dried at 60 °C for 24 hours.

### Preparation of the citrus *Sinensis* activated carbon (AC)

The dried CP powder was placed in crucibles to undergo pyrolysis in a furnace (Wise-Therm, 4 °C min^−1^) at 300 °C for 2 hours. The resulting biochar was washed with deionized water and dried at 100 °C for 2 hours.

The chemical activation was executed by adding 500 mL of H_2_O_2_ (15%) to a 50 g biochar sample and stirring at 25 °C for 24 hours. The resulting AC powder was filtered and dried at 100 °C for 24 hours.

### Adsorbent characterization

The prepared CP and AC adsorbents were characterized using several techniques. The particle size analysis was conducted using Partica LA-950 Laser Diffraction Particle Size Distribution Analyzer-HORIBA. The SEM images were taken on an AIS 2100C microscope (20 kV) with an ASID scanning accessory and EDAX analyzer. The BET analysis was conducted on a Micromeritics ASAP 2010. The EDS was conducted using energy-dispersive X-ray spectroscopy on X'Pert PRO MPD diffractometer. The FTIR was performed on the JASCO FTIR-6300 spectrometer (400–4000 cm^−1^). The zeta potential was measured using Zeta-Meter 4.0.

### Adsorption experiments of metal ions

A batch of sorption experiments of Cu(ii) and Cd(ii) on CP and AC particles was carried out. The experiments were conducted in 50 mL Erlenmeyer flasks containing CuCl_2_ or CdCl_2_·H_2_O (Sigma-Aldrich, analytical grade without further purification) solution with different initial concentrations (100–600 mg L^−1^). Various masses (0.25–2 g) of CP or AC were added at different temperatures (25–100 °C). The initial pH (1–6) was adjusted by adding HNO_3_ (1 M) or NaOH (1 M) solution, and the mixture was continuously stirred. Various samples were then taken at different times (0–180 min) using a micropipette and filtered to 0.45 μm. The filtrate was then analyzed by Atomic Absorption Spectroscopy (AAS; BRAIC spectrophotometer, air–acetylene flame), 324.8 nm wavelength for Cu(ii), and 228.8 nm for Cd(ii) to determine the final concentration of the metal ion.

The adsorption capacity, *Q*_e_ (mg g^−1^), was calculated by applying eqn (1):1
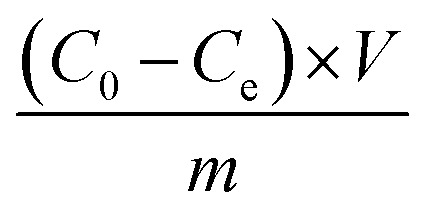
where *C*_0_ and *C*_e_ (mg L^−1^) are the initial and equilibrium metal ion concentration in solution, respectively, *V* (L) is the liquid volume, and *m* (g) is the adsorbent mass.

### Adsorption experiments of methylene blue (MB)

A batch of sorption experiments of MB on CP and AC particles was conducted. The experiments were running in 50 mL Erlenmeyer flasks containing MB solution with different initial concentrations (20–100 mg L^−1^). Various masses (0.25–2 g) of CP or AC were added at different temperatures (25–100 °C), and the mixture was continuously stirred. Various samples were then taken at different times (0–120 min) using a micropipette.

The adsorption capacity, *Q*_e_ (mg g^−1^), was determined by Ultraviolet-Visible spectroscopy (UV-Vis; U-2900, dual-beam spectrometer, 200 V, 664 nm wavelength) by referring to eqn (1), where *C*_0_ and *C*_e_ (mg L^−1^) are the initial and equilibrium MB concentration in solution, respectively.

### Adsorption isotherm models

#### Langmuir isotherm model

Assuming that the adsorbent surface is uniform at a monolayer formed, the adsorption occurs according to the same mechanism, and there are no interactions between the adsorbed molecules; the Langmuir linear eqn (2) can be expressed as:2
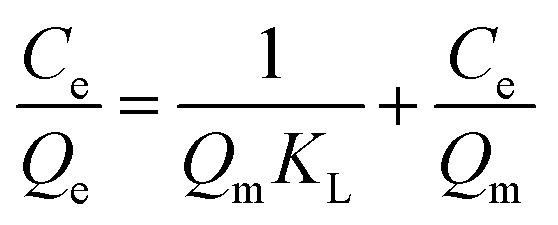
where *C*_e_ is the ions concentration at equilibrium (mg L^−1^), *Q*_e_ is the adsorption capacity at equilibrium (mg g^−1^), *Q*_m_ is the maximum estimated adsorption at monolayer (mg g^−1^), and *K*_L_ is the Langmuir constant linked to the sorption energy (L g^−1^).

Then, *R*_L_, the separation factor constant derived from the Langmuir equation, was calculated as per eqn (3):3
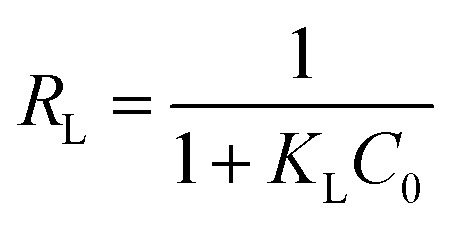
where *C*_0_ is the initial metal ion concentration (mg L^−1^).

#### Freundlich isotherm model

In this model, the adsorption is assumed to form multilayers on a heterogeneous surface. The Freundlich linear equation can be expressed by eqn (4):4
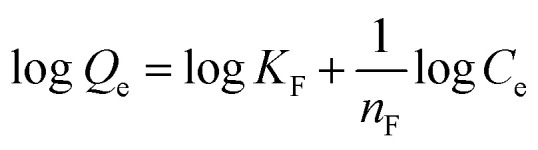
where *Q*_e_ is the adsorption capacity at equilibrium (mg g^−1^), *C*_e_ is the ions concentration at equilibrium (mg L^−1^), *K*_F_ is the Freundlich constant relative to the adsorption capacity, and *n*_F_ is the Freundlich constant.

### Reusability tests

These tests were performed on dried CP and AC samples already loaded by Cu(ii), Cd(ii), and MB at optimum conditions. Each sample loaded with heavy metals was placed in a 150 mL Erlenmeyer flask containing 100 mL of 1 M HCl solution, and the mixture was then stirred at 25 °C for 2 hours. Then, the solution containing the desorbed ions was checked by AAS, and the adsorbent was washed with deionized water and dried at 60 °C for 24 hours. After that, another adsorption experiment was performed on the dried adsorbent at optimum conditions. The same procedure was applied for the CP and AC loaded by MB but with 90% ethanol as eluant. The solutions containing the desorbed MB were investigated using the UV-Vis. 4 reuse cycles of adsorption–desorption were performed (in total, 5 uses of each adsorbent).

All the studies were repeated three times with a standard deviation of 2% and reproducibility of 0.5%.

## Results and discussion

### Adsorbent characterization

#### Particle size analysis

**Fig. 1:**
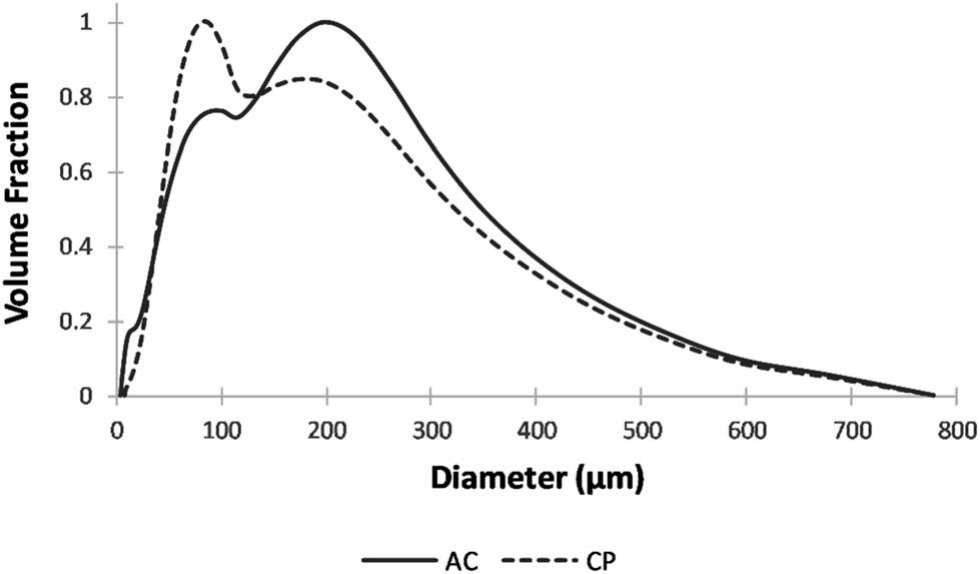
Particle size distribution of CP and AC.

Fig. 1 shows a heterogeneous particle size distribution, ranging from 4 to 780 μm, for CP and AC particles. The size of the majority of CP powdered particles was around 88 μm. However, after carbonization and activation, most AC particles were almost granular with an average diameter of 200 μm.

#### Scanning electron microscopy (SEM)

**Fig. 2:**
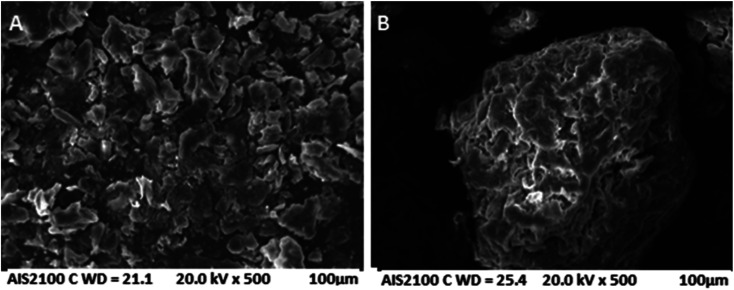
SEM images of (A) CP and (B) AC.

Examination under a scanning electron microscope was used to understand the morphological differences between the CP (Fig. 2A) and AC (Fig. 2B) particles. The SEM images show that both adsorbents have an irregular shape with a rough surface and many pores that can improve the adsorption process. In addition to the decrease in the number of pores, the AC particles appear to agglomerate together (Fig. 2B), confirming the granular size distribution found in the particle size analysis.

#### Brunauer–Emmett–Teller (BET)

The CP and its activated carbon were inspected using the BET technique. The results obtained by N_2_ adsorption–desorption are reported in Table 2. The BET surface area, total pore volume, and average pore size were higher after calcination and H_2_O_2_ activation of the CP precursor. Despite its small surface area, the latter can still be considered a good sorbent due to its average pore size (1.30 nm). Nevertheless, after pyrolysis, the volatile matter content filling the pores and dominating the surface of the original peels is released, yielding an AC adsorbent with a significant pore size and an average BET surface area.^24,25^

**Table 2:** BET data of CP and AC particles

Adsorbent	Specific surface area (m^2^ g^−1^)	Total pore volume (cm^3^ g^−1^)	Average pore size (nm)
CP	1.90	0.130	1.30
AC	96	3.70	12.1

#### Energy-dispersive X-ray analysis (EDS)

EDS analysis has been implemented to investigate the chemical composition present on the surface of the adsorbents used. Table 3 shows the existence of carbon, oxygen, potassium, and calcium elements in both adsorbent types. The carbon element is predominant in both solids with higher content in CP particles. The unexpected lower C content in AC particles can be attributed to the high content of polyphenols components in the citrus peels, which can be volatilized during the pyrolysis process.^26^ In addition, the pyrolysis temperature used in this work (300 °C) can partially degrade the hemicellulose component of the CP upon thermal cracking of the functional groups, more specifically the carbonyl groups.^27,28^

**Table 3:** Elemental analysis of CP and AC particles

Adsorbent	Element	Mass (%)	Atom (%)
CP	C	54.08	71.44
	O	25.19	24.98
	K	1.68	0.68
	Ca	4.34	1.72
AC	C	47.32	64.61
	O	31.9	32.7
	K	1.15	0.48
	Ca	1.75	0.72

#### Fourier transform infrared spectroscopy (FTIR)

**Fig. 3:**
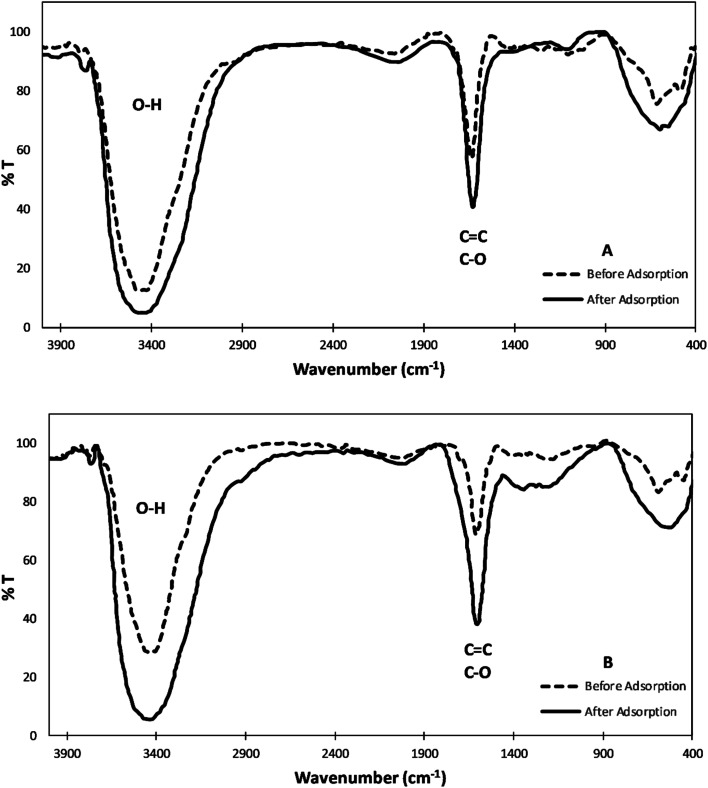
FTIR spectra of (A) CP and (B) AC before and after the adsorption of the heavy metal ions.

Further surface characterization was implemented to investigate the main functional groups present. This characterization method was examined before and after the adsorption of the heavy metal ions. The FTIR spectra show that both CP (Fig. 3A) and AC (Fig. 3B) particles have similar functional groups on their surfaces. Before adsorption, both spectra show a broad and intense band at around 3459 cm^−1^ corresponding to the O–H bond mainly from carboxyls, phenols, and alcohols surface groups and one absorption peak at about 1635 cm^−1^ that may be attributed to the C–O stretching vibration in carbonyl and carboxy groups or –C=C– stretches in the aromatic rings.^29–31^ After adsorption, both FTIR spectra show that the O–H band shifted at around 3440 cm^−1^ which signifies the involvement of the hydroxyl group in binding to the metal ion. Also, the peak emerging at about 1635 cm^−1^ is shifted to 1629 cm^−1^. These slight shifts can be attributed to the surface functional groups' energy changes upon binding to the heavy metals.^4,32^

#### Zeta potential

**Fig. 4:**
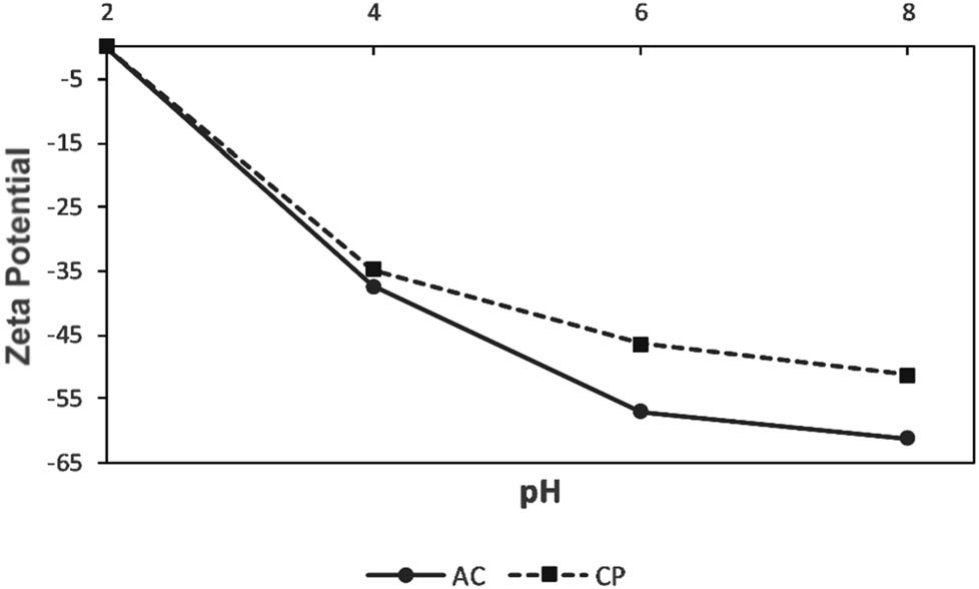
Zeta potentials of CP and AC as a function of pH.

The variation of the zeta potential of the CP and AC particles in terms of pH is shown in Fig. 4. The zeta potential of both adsorbents exhibits negative values at any studied pH (2–8), and it decreases as the pH increases. The highly acidic surface of both solids can improve their ability to bind to positively charged ions in solution.^33^ From pH 4, the zeta potential becomes highly negative, meaning that the colloidal CP and AC adsorbents can show high stability in the solution, preventing their coalescence due to the higher electrostatic repulsion between the particles.^34^

### Adsorption experiments

#### Adsorption of copper and cadmium ions

##### Effect of initial adsorbate concentration and contact time

**Fig. 5:**
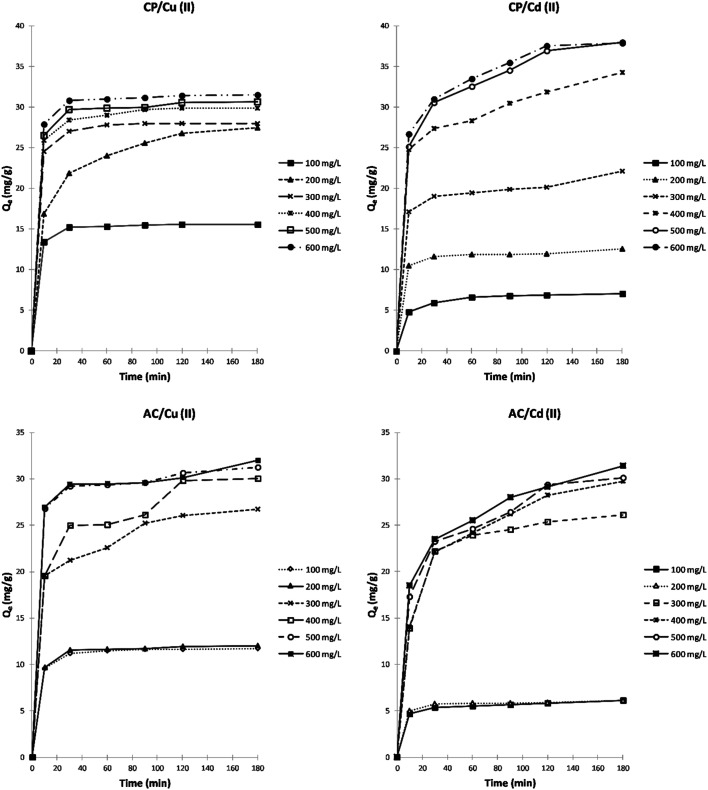
Effect of initial adsorbate (Cu^2+^, Cd^2+^) concentration and contact time on the adsorption capacity (*Q*_e_) of CP and AC particles (1 g of adsorbent, pH 3–4, 25 ± 2 °C).

A comparison between the adsorption capacity of CP and AC in removing Cu(ii) and Cd(ii) ions from water at different initial concentrations and contact times is presented in Fig. 5. The presented data shows that the adsorption capacity (*Q*_e_) increases as the initial adsorbate concentration and contact time increase in all the studied cases. Furthermore, the adsorption capacity of both ions on the CP is slightly higher than the adsorption data obtained on the AC. This may be due to the higher accessibility to the CP pores compared to the AC.

In the case of CP, and after 30 min of contact time, the adsorption capacity reaches almost the maximum and remains constant starting from an initial concentration of 400 mg L^−1^ for Cu^2+^. However, the adsorption capacity for Cd^2+^ keeps rising with increasing initial concentration and time until it reaches almost the maximum after 2 hours at 500 mg L^−1^. On the other hand, the adsorption capacity stays almost constant from 400 mg L^−1^ for Cu^2+^ and Cd^2+^ after 2 hours of stirring using the AC adsorbent. The high ratio of active surface sites to total metal ions in the solution at low concentrations can explain the obtained results; the adsorbent can retain most metal ions and remove them from the water. However, due to the stronger concentration gradient and the greater quantity of ions adsorbed per unit mass of adsorbent, the driving force causes the saturation of the adsorbent. Thus, the ions remain free in the solution.

##### Effect of pH

**Fig. 6:**
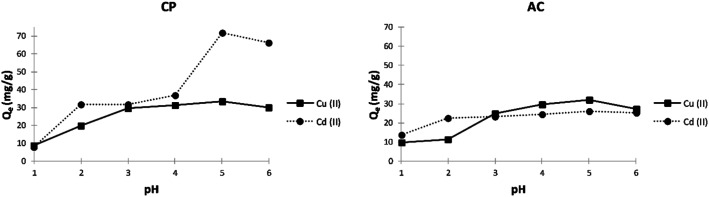
Effect of pH on the adsorption capacity (*Q*_e_) of Cu^2+^ and Cd^2+^ on CP and AC particles (1 g of adsorbent, 400 mg L^−1^ adsorbate initial concentration, 25 ± 2 °C, 2 hours).

The pH of the aqueous solution is a crucial controlling parameter in the adsorption process. It directly influences the adsorbent surface charge and the ionic nature of the metal species. The results of the initial pH effect on the adsorption of both metal ions by CP and AC are presented in Fig. 6. It shows that the adsorption capacity increases as the pH becomes more basic. The maximum adsorption capacity was obtained at pH 5, where 33.3 mg g^−1^ of Cu^2+^ and 71.7 mg g^−1^ of Cd^2+^ were adsorbed by CP. Moreover, a slightly lower maximum adsorption capacity was obtained at pH 5 by the AC particles for both metal ions.

The low adsorption capacities obtained at lower pH values can be attributed to the higher protonation present at the adsorbent surface. Higher pH values yield more significant metal adsorption due to fewer protons, more substantial negative ligands on the surface, and decreased competition between the H^+^ and metal cations.^35^

At higher pH (>5), the insoluble copper and cadmium hydroxide salts begin to precipitate in solution in addition to the adsorption mechanism.^36,37^

##### Effect of adsorbent mass

**Fig. 7:**
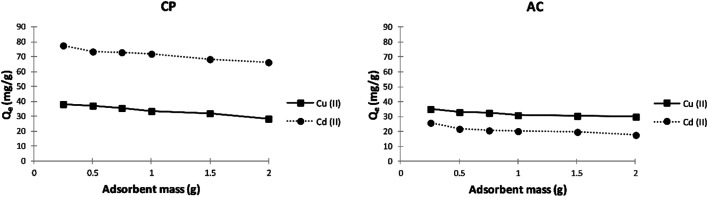
Effect of adsorbent mass on the adsorption capacity (*Q*_e_) of Cu^2+^ and Cd^2+^ on CP and AC particles (400 mg L^−1^ adsorbate initial concentration, pH 5 ± 0.1, 25 ± 2 °C, 2 hours).

For both metal ions and using any of the studied adsorbents, Fig. 7 shows that the adsorption capacity decreases with increasing the mass of the dried powder. These capacities drop is assigned to the rise in unsaturated adsorption sites accompanying the relatively large surface area of the solids, which promotes lowering the mass of ions adsorbed relative to the initial mass of dried adsorbent.

##### Effect of temperature

**Fig. 8:**
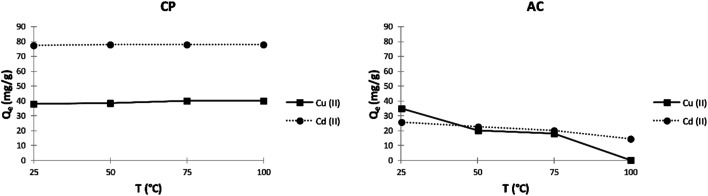
Effect of temperature on the adsorption capacity (*Q*_e_) of Cu^2+^ and Cd^2+^ on CP and AC particles (400 mg L^−1^ adsorbate initial concentration, 0.25 g adsorbent, pH 5 ± 0.1, 2 hours).

The adsorption of Cu^2+^ and Cd^2+^ by the different studied adsorbents was subjected to various temperatures by setting up all other parameters. As shown in Fig. 8, the adsorption capacity of copper ions on the CP slightly increases with increasing the temperature until it reaches a maximum value of 40.1 mg g^−1^ at 75 °C. This slight rise in adsorption capacity can be linked to the increasing number of functional groups on the biomass surface resulting from the higher hydrolysis rate. However, it can be seen in Fig. 8 that the temperature has almost no significance on the adsorption of cadmium ions on CP, and it slightly increases from 77.4 mg g^−1^ to 78 mg g^−1^ at 25 °C and 100 °C, respectively. On the other hand, Fig. 8 shows that the adsorption of Cu^2+^ and Cd^2+^ on the AC particles lessens with increasing the temperature. This can be explained by the fact that the adsorbed species gain enough energy by increasing the temperature so that the desorption rate becomes higher than that of the adsorption.

#### Adsorption isotherm models

Adsorption isothermal investigation was implemented to describe the interaction between the studied metal ions and the two types of adsorbents, CP and AC, and to estimate the maximum adsorption capacity. Both Langmuir and Freundlich models were employed in this analytical investigation.

##### Langmuir isotherm model

**Fig. 9:**
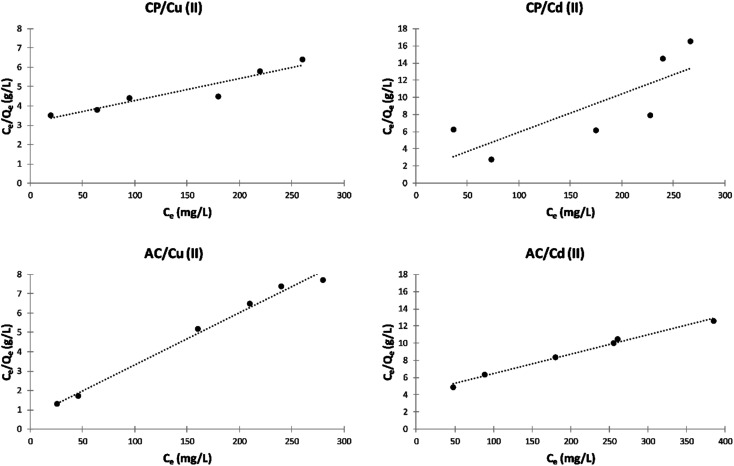
Langmuir isotherm plots of the adsorption of Cu^2+^ and Cd^2+^ on CP and AC particles (0.25 g adsorbent, pH 5 ± 0.1, 25 ± 2 °C, 2 hours).

The Langmuir isotherms of the adsorption of Cu^2+^ and Cd^2+^ on the CP and AC particles are shown in Fig. 9. In addition, the data obtained from the Langmuir isotherms and the *R*_L_ calculated values are presented in Table 4. The *R*_L_ values were used as the primary indicator of the Langmuir isotherm quality. The described sorption is favorable if *R*_L_ lies between 0 and 1.

**Table 4:** Langmuir model data of the adsorption of Cu^2+^ and Cd^2+^ on CP and AC particles

Adsorbate	Adsorbent	*K*_L_	*Q*_m_	*R*_L_	*R* ^2^	Adsorption
Cu(ii)	CP	0.0390	40.00	0.985	0.9889	Favorable
Cu(ii)	AC	0.0068	38.90	0.997	0.9935	Favorable
Cd(ii)	CP	0.0041	80.00	0.997	0.9005	Favorable
Cd(ii)	AC	0.0196	26.88	0.992	0.5623	Favorable

The *R*_L_ values obtained in this study are smaller than 1, indicating that the adsorption is favorable for the four different studied cases.^4^ By comparing the maximum adsorption at monolayer (*Q*_m_) values in Table 4, it can be noticed that the adsorption by CP is more efficient than by its derived AC.

##### Freundlich isotherm model

**Fig. 10:**
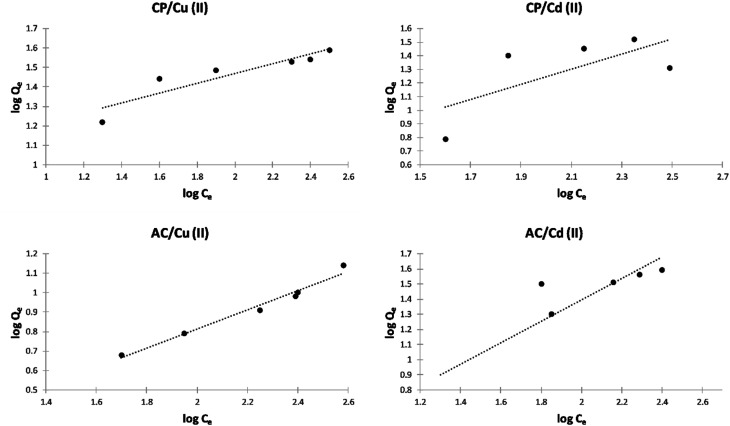
Freundlich isotherm plots of the adsorption of Cu^2+^ and Cd^2+^ on CP and AC particles (0.25 g adsorbent, pH 5 ± 0.1, 25 ± 2 °C, 2 hours).

The Freundlich isotherms of the adsorption of Cu^2+^ and Cd^2+^ on the CP and AC particles are shown in Fig. 10. Meanwhile, the data obtained from the Freundlich isotherms and the *n*_F_ determined values are presented in Table 5. The *n*_F_ values were determined to be used as a primary indicator of the adsorption intensity. In general, a favorable sorption is characterized by 1 < *n*_F_ < 10 and a linear sorption is characterized by *n*_F_ = 1. All the *n*_F_ values determined by applying the Freundlich isotherm model lie between 1 and 10, indicating that the adsorption was favorable either by CP or AC for both metal ions. However, the adsorption of Cu^2+^ on CP particles is the most favored.^4^

**Table 5:** Freundlich model data of the adsorption of Cu^2+^ and Cd^2+^ on CP and AC particles

Adsorbate	Adsorbent	*K*_F_	*n*_F_	*R* ^2^
Cu(ii)	CP	8.6200	3.595	0.8462
Cu(ii)	AC	0.6860	2.058	0.9723
Cd(ii)	CP	0.9641	1.417	0.8144
Cd(ii)	AC	1.2290	1.842	0.4863

Finally, by comparing the correlation coefficients (*R*^2^) resulting from the Langmuir (Table 4) and Freundlich (Table 5) isotherm models, it can be noticed that the Langmuir model is more applicable for the adsorption of both cations on CP and AC, forming a monolayer. This may be due to a homogeneous distribution of active sites on the adsorbents' surface.^29^

#### Adsorption of methylene blue (MB)

After building up the calibration curve of MB by UV-Vis spectroscopy at wavelength 664 nm, the initial adsorbent mass and temperature effects on the adsorption of MB in water were studied and yielded almost the same results obtained in the adsorption tests of the metal ions. Thus, the following study was conducted at 25 °C by employing 0.25 g of adsorbent.

##### Effect of initial adsorbate concentration and contact time

**Fig. 11:**
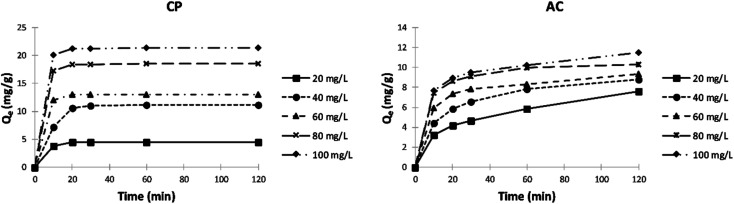
Effect of initial MB concentration and contact time on the adsorption capacity (*Q*_e_) of CP and AC particles (0.25 g adsorbent, 25 ± 2 °C, 2 hours).

Fig. 11 shows the variation of the adsorption capacity as a function of contact time with both types of adsorbent using various initial concentrations of MB. The adsorption capacity of CP particles increases by increasing the initial MB concentration and reaching the maximum after 20 min. However, the adsorption capacity keeps rising with MB concentration and time using the AC adsorbent. By comparing the performance of both adsorbents, it can be noticed that the adsorption capacity and rate are higher using CP.

#### Adsorption isotherm models

##### Langmuir isotherm model

**Fig. 12:**
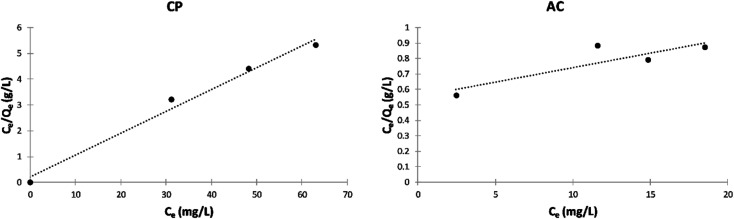
Langmuir isotherm plots of the adsorption of MB on CP and AC particles (0.25 g adsorbent, 25 ± 2 °C, 2 hours).

Fig. 12 shows the Langmuir isotherms of the adsorption of MB by CP and AC particles. The calculated separation factor (*R*_L_) values in Table 6 elucidate that the adsorption is favorable in both cases since they lie between 0 and 1.

**Table 6:** Langmuir model data of the adsorption of MB on CP and AC particles

Adsorbate	Adsorbent	*K*_L_	*Q*_m_	*R*_L_	*R* ^2^	Adsorption
MB	CP	0.031	56.81	0.997	0.752	Favorable
MB	AC	0.400	11.36	0.961	0.985	Favorable

##### Freundlich isotherm model

**Fig. 13:**
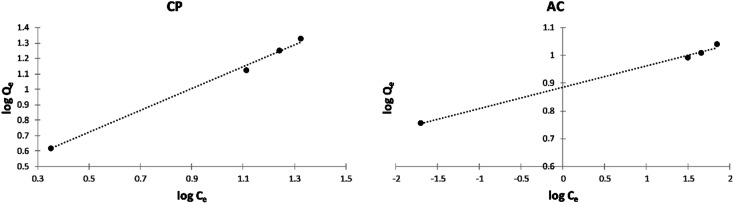
Freundlich isotherm plots of the adsorption of MB on CP and AC particles (0.25 g adsorbent, 25 ± 2 °C, 2 hours).

The Freundlich isotherms of the adsorption of MB on the CP and AC particles shown in Fig. 13, and their related data and the *n*_F_ determined values shown in Table 7 indicate that the adsorption was favorable for both solids, especially for CP.

**Table 7:** Freundlich model data of the adsorption of MB on CP and AC particles

Adsorbate	Adsorbent	*K*_F_	*n*_F_	*R* ^2^
MB	CP	2.070	1.266	0.9931
MB	AC	7.379	10.99	0.9960

Furthermore, the correlation coefficients (*R*^2^) yielding from the Langmuir (Table 6) and Freundlich (Table 7) isotherm models designate that the Freundlich model is preferred for the adsorption of MB on CP and AC. This can be attributed to the initial sites saturation resulting from stronger adsorption of MB, and then the adsorption strength decreases when the adsorbent's site occupation increases.^38^

### Reusability tests

**Fig. 14:**
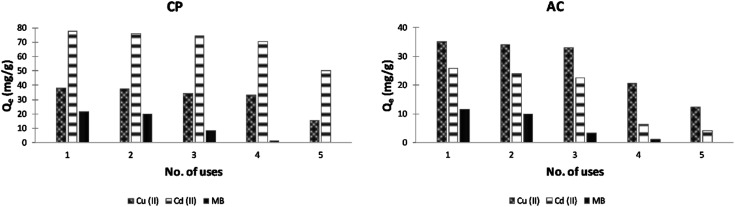
Plots of adsorption capacity of Cu(ii), Cd(ii), and MB *versus* no. of uses of CP and AC adsorbents (optimum conditions).

The use of adsorption in treating wastewater has a significant advantage over the traditional methods since it allows adsorbent regeneration. A reuse study was carried out in this work by exposing the used CP and AC adsorbents (considered as the first use cycle) to 2 different eluents up to 4 reuse cycles; 1 M HCl solution was used in the desorption of heavy metals, and ethanol solvent was implemented for the MB. As illustrated in Fig. 14, the CP loses almost 13% and 9% only of its original adsorption capacity for Cu and Cd ions, respectively, after 3 reuse cycles (4 uses in total). However, it cannot be used more than 2 times to remove MB from water since it loses around 43% of its adsorption capacity starting from the third use.

In the case of AC, only 2 reuse cycles are recommended since the adsorbent loses 41 to 75% of its original adsorption capacity for heavy metals after the fourth use. For the MB, similar to the CP results were obtained.

## Conclusions

This work highlighted the main characteristics of the low-cost biosorbents, citrus *Sinensis* fruit peel and its activated carbon, and their potentialities and efficiencies in removing Cu and Cd ions and MB from water. The prepared porous solids, presenting hydroxyl surface groups, showed optimum adsorption for organic and inorganic species using 0.25 g of adsorbent at pH 5 (for metal ions only) and 25 °C. The optimum dosage of the initial adsorbate was 400 mg L^−1^ for metal ions and 100 mg L^−1^ for methylene blue with a 2 h contact time needed. In general, in most adsorption studies, the removal process was fast in the early stages, then became slower to reach a maximum. Langmuir and Freundlich's isotherms indicated that the metal ions were adsorbed at a monolayer, covering a concentration range from 20 to 267 mg L^−1^. In comparison, heterogeneous adsorption was expectedly occurring for the MB molecules covering a concentration range of up to 100 mg L^−1^. Furthermore, CP showed slightly better adsorption capacity than activated carbon in removing the studied organic and inorganic species. Good reuse performance was exhibited by both adsorbents that can be used up to 2–4 times depending on the nature of the adsorbent and contaminants. Finally, this study positively impacts controlling water contamination, leading to the subsequent assessment of the removal efficiency of the CP and its AC from natural wastewater and industrial effluents treatment.

## Author contributions

Conceptualization, formal analysis, methodology, and supervision: WEM, AH, MM, and MR; investigation and validation: ZAK and ZY; funding acquisition and project administration: AH and MR; writing and editing: WEM, ZAK, and ZY.

## Conflicts of interest

There are no conflicts to declare.

## Supplementary Material
